# Disparities in neighbourhood characteristics and 10-year dementia risk by nativity status

**DOI:** 10.1017/S2045796024000076

**Published:** 2024-02-15

**Authors:** R. Wong, D. Soong

**Affiliations:** 1 Department of Public Health and Preventive Medicine, Norton College of Medicine, SUNY Upstate Medical University, Syracuse, NY, USA; 2 Department of Geriatrics, SUNY Upstate Medical University, Syracuse, NY, USA; 3 Norton College of Medicine, SUNY Upstate Medical University, Syracuse, NY, USA

**Keywords:** aging, dementia, disparities, environment, immigrant, nativity, neighbourhood, older adult, physical disorder, social cohesion

## Abstract

**Aims:**

Prior research indicates that neighbourhood disadvantage increases dementia risk. There is, however, inconclusive evidence on the relationship between nativity and cognitive impairment. To our knowledge, our study is the first to analyse how nativity and neighbourhood interact to influence dementia risk.

**Methods:**

Ten years of prospective cohort data (2011–2020) were retrieved from the National Health and Aging Trends Study, a nationally representative sample of 5,362 U.S. older adults aged 65+. Cox regression analysed time to dementia diagnosis using nativity status (foreign- or native-born) and composite scores for neighbourhood physical disorder (litter, graffiti and vacancies) and social cohesion (know, help and trust each other), after applying sampling weights and imputing missing data.

**Results:**

In a weighted sample representing 26.9 million older adults, about 9.5% (*n* = 2.5 million) identified as foreign-born and 24.4% (*n* = 6.5 million) had an incident dementia diagnosis. Average baseline neighbourhood physical disorder was 0.19 (range 0–9), and baseline social cohesion was 4.28 (range 0–6). Baseline neighbourhood physical disorder was significantly higher among foreign-born (mean = 0.28) compared to native-born (mean = 0.18) older adults (*t* = −2.4, *p* = .02). Baseline neighbourhood social cohesion was significantly lower for foreign-born (mean = 3.57) compared to native-born (mean = 4.33) older adults (*t* = 5.5, *p* < .001). After adjusting for sociodemographic, health and neighbourhood variables, foreign-born older adults had a 51% significantly higher dementia risk (adjusted hazard ratio = 1.51, 95% CI = 1.19–1.90, *p* < .01). There were no significant interactions for nativity with neighbourhood physical disorder or social cohesion.

**Conclusions:**

Our findings suggest that foreign-born older adults have higher neighbourhood physical disorder and lower social cohesion compared to native-born older adults. Despite the higher dementia risk, we observed for foreign-born older adults, and this relationship was not moderated by either neighbourhood physical disorder or social cohesion. Further research is needed to understand what factors are contributing to elevated dementia risk among foreign-born older adults.

## Introduction

Previous research has shown a relationship between neighbourhood characteristics and cognitive health among older adults. One study of older adults in the United States (U.S.) found that greater access to parks and greenspaces was independently associated with a 4% significantly greater odds of maintained cognitive function scores over 6 years (Besser *et al.*, 2017). In contrast, neighbourhood physical disorder at baseline and follow-up was associated with a 10% and 11% increased dementia risk, respectively, during a 9-year window using data from a national U.S. older adult sample (Wong and Wang, 2022). Ultimately, these varying neighbourhood characteristics may influence older adults’ motivation to venture outdoors, thereby altering opportunities for exercise and socialization, both of which are known to aid in maintaining cognitive function during the aging process (Guo *et al.*, 2021).

Immigrants (e.g. adults who are foreign-born) often choose to reside in ethnic enclaves, which can potentially offer protective effects against cognitive decline. These enclaves facilitate stronger social connections, a greater sense of community and a supportive environment (Guo *et al.*, 2021). However, it is important to note that ethnic enclaves may also be situated in neighbourhoods with higher perceived disorder, characterized by issues such as vandalism, broken curbs and unsafe traffic conditions (Guo *et al.*, 2021). These environmental factors can contribute to increased stress levels and potentially exacerbate cognitive conditions (Wong and Wang, 2022).

While new U.S. immigration rates have gradually slowed down over the past decade, the overall immigrant population has continued to grow steadily (Ward and Batalova, 2023). Notably, the most recent 2020 U.S. Census recorded a historic figure of about 45 million foreign-born individuals in the U.S. (Congressional Budget Office, 2023). Among this population, there are 7.5 million immigrants aged 60 and older, and this number is projected to rise to 20 million by 2050 (Camarota and Zeigler, 2021; Mizoguchi *et al.*, 2019). Furthermore, by the year 2060, foreign-born immigrants are estimated to account for nearly a quarter of the U.S. older adult population (Mizoguchi *et al.*, 2019).

There are inconclusive findings regarding differences in cognitive impairment risk between foreign-born and native-born groups (Xu *et al.*, 2017). Several studies indicate that nativity is not associated with cognitive function. In a cross-sectional sample of older Asian Americans and dementia cases in California, nativity was not shown to change incidence rates for dementia (Hayes-Larson *et al.*, 2023). Similarly, a longitudinal study comparing native and foreign-born refugee residents aged 40 years and older in Denmark did not find a relationship between neighbourhood disadvantage and dementia risk (Kim *et al.*, 2022).

Other studies propose that foreign-born individuals may benefit from the ‘healthy immigrant effect’, which suggests that wealthier individuals with more resources are more likely to immigrate, resulting in better health outcomes for immigrants (Markides and Rote, 2019). One study found that the healthy immigrant effect extends to include cognitive benefits in older Mexican Americans (Hill *et al.*, 2012). Specifically, for those that immigrated between the ages of 20 and 49, immigrants saw a slower decline in cognitive function scores (Hill *et al.*, 2012).

More recent studies have demonstrated that the healthy immigrant effect diminishes over time due to inherent stresses associated with immigration, such as acculturation, labour-intensive jobs and language barriers, which impede social engagement and access to resources (Markides and Rote, 2019). The cumulative impact of these stresses ultimately has detrimental effects. One study revealed that older immigrants had a 70% higher odds of dementia compared to their native-born U.S. counterparts, after adjusting for demographic, socio-economic and health factors (Franco and Choi, 2020). Further, except for non-Hispanic Black immigrants, those who identified as non-Hispanic White, Hispanic and of other racial and ethnic groups had a higher prevalence of dementia when compared to native-born individuals (Moon *et al.*, 2019).

Limited research has been conducted on the neighbourhood-cognition relationship between foreign-born and native-born residents. The available studies are limited in scope to a single racial or ethnic group. For example, residing in an ethnic enclave was found to have a protective effect against cognitive decline for foreign-born older Mexican Americans (Weden *et al.*, 2017). Conversely, a more recent study revealed that within U.S. Chinese American populations, living in a Chinatown was a risk factor for poorer cognitive function (Guo *et al.*, 2021). These inconsistent findings limit generalizability to the broader U.S. population and make it challenging to draw a definitive conclusion regarding the relationship between neighbourhood, nativity and cognition.

Furthermore, the current literature exhibits additional limitations and constraints. Many studies have relied on cross-sectional data, which hinder the ability to establish causal relationships between neighbourhood characteristics and the progression of a disease like dementia (Besser *et al.*, 2017; Franco and Choi, 2020; Guo *et al.*, 2021; Jia *et al.*, 2020; Moon *et al.*, 2019; Pase *et al.*, 2022). Moreover, previous research has focused on cognitive impairment rather than dementia itself, creating a gap in knowledge regarding the specific effects of neighbourhood and nativity on dementia onset (Guo *et al.*, 2021; Hill *et al.*, 2012; Jia *et al.*, 2020; Weden *et al.*, 2017). These limitations highlight the need for longitudinal studies that specifically investigate the relationship between neighbourhood factors, nativity and dementia progression.

Our present study addresses these gaps and limitations by analysing 10 years of prospective longitudinal data. To the best of our knowledge, our study is the first to examine how neighbourhood characteristics interact with nativity to influence dementia risk using a national U.S. older adult sample. Our specific objectives include the following: (1) examine how nativity status is associated with dementia risk among older adults, (2) examine differences in neighbourhood physical disorder and social cohesion by nativity status among older adults and (3) examine how neighbourhood moderates the relationship between nativity status and dementia risk among older adults.

## Methods

### Data source

Data were retrieved from the National Health and Aging Trends Study (NHATS), a longitudinal panel study that surveys a nationally representative sample of Medicare beneficiaries aged 65 years and older within the U.S. Ten annual waves between 2011 and 2020 of the NHATS were analysed for this study. Full details on NHATS data collection procedures and sampling methods are described elsewhere (Freedman *et al.*, 2022). From the initial sample of 8,245 respondents in the 2011 NHATS cohort, we sequentially excluded respondents who were not community-dwelling (*n* = 1,048) and had a pre-existing dementia diagnosis (*n* = 1,835). Therefore, our final sample included 5,362 community-dwelling older adults at baseline without a prior dementia diagnosis, described later below.

### Nativity measure

The nativity variable was derived from the question, ‘Were you born in the U.S.?’. A ‘yes’ response was coded as native-born, and a ‘no’ response was coded as foreign-born.

### Neighbourhood measures

Neighbourhood characteristics were physical disorder and social cohesion, which were each measured with separate composite scores. These composite scores were developed in the same approach used previously in a separate study using the NHATS data (Wong and Wang, 2022) and similar to the Population Study of Chinese Elderly data (Guo *et al.*, 2021), which revealed sufficient validity for both neighbourhood physical disorder (*α* = 0.80) and social cohesion (*α* = 0.86). The neighbourhood physical disorder composite score was developed from a three-item home environment questionnaire: litter, graffiti and vacancies. For each item, the NHATS interviewer was asked to rate their agreement (none, a little, some or a lot) when standing in front of the respondent’s home/building and looking around in every direction. Litter was defined as ‘Litter, broken glass or trash on sidewalks and streets’. Graffiti was defined as ‘Graffiti on buildings and walls’. Vacancies were defined as ‘Vacant or deserted houses or storefronts’. After combining these three measures, physical disorder composite scores ranged from 0 to 9, with higher values indicating neighbourhoods with more physical disorder.

Similarly, the neighbourhood social cohesion composite score was developed from a three-item community environment questionnaire: knowing, helping and trusting. For each item, respondents were asked to rate their agreement (do not agree, agree a little or agree a lot). Knowing was asked as ‘People in this community know each other very well’. Helping was asked as ‘People in this community are willing to help each other’. Trusting was asked as ‘People in this community can be trusted’. After combining these three measures, social cohesion composite scores ranged from 0 to 6, with higher values indicating neighbourhoods with more social cohesion.

Similar to the approach used previously from a separate study using neighbourhood measures in the NHATS data (Wong and Wang, 2022), we constructed composite scores for physical disorder and social cohesion at baseline (2011) and follow-up (year before death, drop-out or dementia). For both the physical disorder and social cohesion variables, a change variable was also computed by subtracting the follow-up score from the baseline score. Therefore, physical disorder change had a range from −9 to +9, with higher values indicating more disorder at follow-up compared to baseline. Likewise, social cohesion change had a range from −6 to +6, with higher values indicating more cohesion at follow-up compared to baseline. The regression models included only the baseline and change composite scores to limit multicollinearity due to a high correlation between follow-up and change scores.

### Dementia measure

The dependent variable was incident dementia diagnosis, which occurred during the period between 2012 and 2020, after the exclusion of pre-existing dementia diagnoses at baseline (2011). Dementia diagnosis was derived from an NHATS algorithm (Kasper *et al.*, 2013) that uses three cognitive measures: (1) AD8 Dementia Screening Interview, (2) cognitive tests that evaluate the respondents’ memory (e.g. immediate 10-word recall), orientation (e.g. date) and executive function (e.g. clock drawing test) and (3) self-report of an Alzheimer’s or dementia diagnosis by a doctor. From these cognitive measures, the NHATS algorithm generated a binary dementia diagnosis (probable or possible), which has been validated to have a high sensitivity of 85.7% and high specificity of 83.7% (Kasper *et al.*, 2013).

### Covariates

Regression models were adjusted for sociodemographic and health variables. Sociodemographic variables included age, sex (male or female), race and ethnicity (non-Hispanic White, non-Hispanic Black, Hispanic, Asian or Other), highest level of education (less than high school, high school or college), total income, marital status (married or unmarried) and metropolitan residence (metro or non-metro). Health variables included self-rated health (poor to excellent), body mass index (BMI), limitations for activities of daily living (no ADL limitations or at least one ADL limitation), proxy respondent, major depressive disorder, generalized anxiety disorder, heart attack history, hypertension history, diabetes history and stroke history.

### Analysis plan

Descriptive statistics for continuous variables were reported as mean and standard deviation, whereas categorical variables were reported as frequencies and proportions. Differences in average neighbourhood physical disorder and social cohesion by nativity status were analysed using one-way analysis of variance (ANOVA) tests. We used Cox proportional hazards models to analyse time (number of years) to dementia diagnosis starting from baseline (2011). All regression models applied complex survey sampling weights and adjusted for sociodemographics and health. About 15% of the sample was lost through listwise deletion. To maximize the full number of respondents in the data set and minimize bias due to missing data, we used multiple imputation by chained equation (MICE) to generate 15 imputed data files with 10 iterations each. We performed a sensitivity analysis comparing results from MICE versus listwise deletion. Statistical analyses were performed in Stata 18 with two-tailed tests at a 0.05 significance level.

## Results

### Sample characteristics

After applying survey sampling weights, the 5,362 NHATS respondents represented 26,851,237 older adults in the U.S. population. Among the respondents, 9.5% (*n* = 2.5 million) identified as foreign-born in the weighted sample, which is comparable to 9.3% (*n* = 492) in the unweighted sample. Additionally, approximately 24.4% (*n* = 6.5 million) of the respondents had an incident dementia diagnosis between 2012 and 2020. The mean age of the sample population was 73.8 years. The majority of the respondents were female (56.2%), non-Hispanic White (84.1%), married (61.7%) and resided in a metropolitan area (81.4%). On average, respondents reported a yearly income of about $64,000.

The native-born population primarily consisted of non-Hispanic White individuals (88.8%), whereas the foreign-born population was more diverse, with a higher representation of Hispanic (36%) and Asian (16%) older adults (*F* = 286.47, *p* < .001). Foreign-born individuals were in general significantly more likely than native-born individuals to have lower levels of education (*F* = 15.15, *p* < .001). Moreover, a higher proportion of foreign-born individuals (93.3%) resided in metropolitan areas compared to native-born individuals (80.1%) (*F* = 9.08, *p* < .01).

As indicated in Table 1, most respondents rated their health as good. Only a minority reported limitations in ADL (1.4%), had a proxy respondent (1.4%) or experienced depression (10.9%), anxiety (9.9%), diabetes (22.2%), heart attack (12.3%) or stroke (9.8%). However, a substantial proportion of respondents had hypertension (63.4%) and were overweight, with an average BMI of 27.9.

**Table 1.** Weighted sample characteristics^a^

	Whole sample (*n* = 26.6)	Native-born (90.5%, *n* = 24.1)	Foreign-born (9.5%, *n* = 2.5)	Bivariate test^b^
Dementia diagnosis	24.4% (6.5)	23.3% (5.6)	34.8% (0.9)	*F* = 33.52, <.001
**Sociodemographics**				
Age (mean, SD)	73.8 (6.5)	73.9 (6.5)	73.5 (6.5)	*t* = 0.88, .38
Female	56.2% (15.0)	56.3% (14.0)	55.1% (1.4)	*F* = 0.13, .72
Race and ethnicity				*F* = 286.47, <.001
White, non-Hispanic	84.1% (22.0)	88.8% (21.0)	39.2% (1.0)	
Black, non-Hispanic	7.4% (2.0)	7.4% (1.8)	7.6% (0.2)	
Hispanic	5.5% (1.5)	2.3% (0.6)	36.0% (0.9)	
Asian	1.8% (0.5)	0.4% (0.1)	16.0% (0.4)	
Other	1.2% (0.3)	1.2% (0.3)	1.2% (0.03)	
Highest level of education				*F* = 15.15, <.001
<High school	8.6% (2.3)	7.9% (1.9)	14.6% (0.4)	
High school degree	27.6% (7.3)	28.7% (6.9)	17.2% (0.4)	
College degree	63.8% (17.0)	63.4% (15.0)	68.2% (1.7)	
Income (thousands)	64.0 (188.3)	65.2 (181.9)	54.6 (245.6)	*t* = 0.60, .55
Married	61.7% (16.0)	61.5% (15.0)	64.0% (1.6)	*F* = 0.58, .45
Metropolitan residence	81.4% (22.0)	80.1% (19.0)	93.3% (2.4)	*F* = 9.08, <.01
**Health**				
Self-rated health (0−4; poor–excellent) (mean, SD)	2.4 (1.1)	2.4 (1.1)	2.2 (1.1)	*t* = 4.14, <.001
Body mass index	27.9 (5.5)	28.0 (5.6)	27.0 (4.7)	*t* = 4.17, <.001
ADL limitations	7.5% (2.0)	7.0% (1.7)	12.3% (0.3)	*F* = 19.98, <.001
Proxy respondent	1.4% (0.4)	0.9% (0.2)	5.5% (0.1)	*F* = 41.90, <.001
Depression	10.9% (2.9)	10.5% (2.5)	15.8% (0.4)	*F* = 5.93, .02
Anxiety	9.9% (2.6)	9.7% (2.3)	11.7% (0.29)	*F* = 1.81, .18
Heart attack	12.3% (3.3)	12.6% (3.0)	9.8% (0.25)	*F* = 2.86, .10
Hypertension	63.4% (17.0)	63.6% (15.0)	61.7% (1.6)	*F* = 0.96, .33
Diabetes	22.2% (5.9)	22.1% (5.3)	22.4% (0.6)	*F* = 0.02, .89
Stroke	7.8% (2.1)	7.9% (1.9)	6.3% (0.2)	*F* = 1.66, .02
**Physical disorder (mean, SD)^c^**				
Baseline	0.19 (0.7)	0.18 (0.6)	0.28 (0.8)	*t* = −2.40, .02
Follow-up	0.15 (0.6)	0.15 (0.6)	0.24 (0.7)	*t* = −3.26, <.01
Change	−0.03 (0.6)	−0.03 (0.6)	−0.01 (0.6)	*t* = −0.69, .49
**Social cohesion (mean, SD)^d^**				
Baseline	4.28 (1.6)	4.33 (1.6)	3.75 (1.7)	*t* = 5.50, <.001
Follow-up	4.32 (1.6)	4.37 (1.6)	3.85 (1.7)	*t* = 4.68, <.001
Change	0.03 (1.3)	0.02 (1.3)	0.10 (1.4)	*t* = −1.08, .27

a Unless otherwise indicated, data are expressed as percent and frequency (in millions).

b *T*-test for continuous variables and chi-square for categorical variables.

c Physical disorder at baseline and follow-up has a range from 0 to 9, with higher values indicating more disorder. Physical disorder change has a range from −9 to +9, with higher values indicating more disorder at follow-up compared to baseline. Follow-up refers to year before death, drop-out or dementia.

d Social cohesion at baseline and follow-up has a range from 0 to 6, with higher values indicating more cohesion. Social cohesion change has a range from −6 to +6, with higher values indicating more cohesion at follow-up compared to baseline. Follow-up refers to year before death, drop-out or dementia.

Significant differences were observed between foreign-born and native-born individuals in several health indicators. Foreign-born individuals were significantly more likely to have ADL limitations (*F* = 19.98, *p* < .001), a proxy respondent (*F* = 41.90, *p* < .001) and depression (*F* = 5.93, *p* < .02) compared to native-born individuals. However, they exhibited a lower prevalence of previous strokes (*F* = 1.66, *p* = .02) and lower average BMI (*t* = 4.17, *p* < .001).

### Neighbourhood differences by nativity

The relationship between nativity and neighbourhood characteristics was examined at both baseline and follow-up (Table 1). Physical disorder was significantly higher among foreign-born individuals compared to native-born individuals at both baseline (*t* = −2.40, *p* = .02) and follow-up (*t* = −3.26, *p* < .01). Social cohesion, conversely, was significantly lower among foreign-born individuals compared to native-born individuals at both baseline (*t* = 5.50, *p* < .001) and follow-up (*t* = 4.68, *p* < .001). However, when examining the change between baseline and follow-up for both physical disorder and social cohesion, no statistically significant differences were found after stratifying by nativity.

### Cox regression results

Our unadjusted crude model indicated that foreign-born status was significantly associated with an 82% increased dementia risk (hazard ratio = 1.82, 95% CI = 1.60–2.08, *p* < .001) (Table 2, Model A). After adjusting for sociodemographics and health, foreign-born older adults maintained a 49% significant increased dementia risk (adjusted hazard ratio [aHR] = 1.49, 95% CI = 1.19–1.87, *p* < .01) (Table 2, Model B). In our fully-adjusted model, nativity status was again significantly associated with dementia diagnosis, with those who were foreign-born having a 51% higher increased risk (aHR = 1.51, 95% CI = 1.19–1.90, *p* < .01).

**Table 2.** Weighted cox regression of nativity and neighbourhood on dementia risk

	Model A Hazard ratio (95% CI), *p*	Model B Hazard ratio (95% CI), *p*	Model C Hazard ratio (95% CI), *p*	Model D Hazard ratio (95% CI), *p*
**Nativity**				
Native-born	Reference	Reference	Reference	Reference
Foreign-born	1.82 (1.60−2.08), <.001	1.49 (1.19−1.87), <.01	1.51 (1.19, 1.90), <.01	1.70 (1.01, 2.87), .04
**Sociodemographics**				
Age		1.08 (1.07−1.09), <.001	1.08 (1.07, 1.09), <.001	1.08 (1.07, 1.09), <.001
Female		0.83 (0.72−0.94), <.01	0.84 (0.73, 0.95), <.01	0.83 (0.73, 0.95), <.01
Race and ethnicity				
White, non-Hispanic		Reference	Reference	Reference
Black, non-Hispanic		1.57 (1.34−1.84), <.001	1.49 (1.27, 1.76), <.001	1.47 (1.24, 1.75), <.001
Hispanic		1.27 (0.97−1.66), .08	1.20 (0.92, 1.58), .17	1.23 (0.94, 1.62), .13
Asian, non-Hispanic		0.93 (0.61−1.42), .72	0.94 (0.62, 1.44), .78	0.93 (0.60, 1.42), .72
Other		1.18 (0.79−1.78), .41	1.13 (0.74, 1.74), .57	1.11 (0.72, 1.73), .62
Highest level of education				
<High school		Reference	Reference	Reference
High school degree		0.71 (0.62−0.81), <.001	0.72 (0.63, 0.83), <.001	0.72 (0.63, 0.82), <.001
College degree		0.57 (0.50−0.65), <.001	0.58 (0.51, 0.66), <.001	0.58 (0.51, 0.66), <.001
Total income (log)		0.97 (0.93−1.01), .13	0.97 (0.94, 1.02), .21	0.98 (0.94, 1.02), .22
Married		0.89 (0.78−1.01), .08	0.89 (0.78, 1.02), .08	0.89 (0.78, 1.02), .09
Metropolitan residence		0.83 (0.71−0.98), .03	0.83 (0.71, 0.98), .03	0.83 (0.71, 0.98), .03
**Health**				
Self-rated health (0−4; poor–excellent)		0.89 (0.84−0.94), <.001	0.89 (0.84, 0.94), <.001	0.89 (0.84, 0.94), <.001
Body mass index		0.99 (0.98−1.00), .21	0.99 (0.98, 1.00), .21	0.99 (0.98, 1.00), .22
ADL limitations		1.34 (1.15−1.56), <.001	1.34 (1.16, 1.56), <.001	1.34 (1.15, 1.56), <.001
Proxy respondent		2.18 (1.57−3.03), <.001	2.16 (1.54, 3.03), <.001	2.14 (1.52, 3.02), <.001
Depression		1.25 (1.04−1.51), .02	1.26 (1.05, 1.51), .01	1.26 (1.05, 1.51), .01
Anxiety		1.40 (1.14−1.71), <.01	1.41 (1.14, 1.73), <.01	1.41 (1.14, 1.73), <.01
Heart attack		1.19 (1.03−1.38), .02	1.20 (1.04, 1.39), .02	1.19 (1.03, 1.39), .02
Hypertension		0.94 (0.84−1.06), .30	0.94 (0.84, 1.05), .30	0.94 (0.85, 1.05), .30
Diabetes		1.23 (1.08−1.40), <.01	1.23 (1.08, 1.41), <.01	1.23 (1.08, 1.40), <.01
Stroke		1.03 (0.86−1.23), .73	1.02 (0.85, 1.22), .81	1.01 (0.85, 1.21), .88
**Neighbourhood**				
Physical disorder baseline			1.15 (1.05, 1.25), <.001	1.18 (1.08, 1.30), <.01
Physical disorder change			1.14 (1.06, 1.22), <.01	1.16 (1.08, 1.25), <.001
Social cohesion baseline			1.02 (0.98, 1.06), .31	1.02 (0.98, 1.07), .32
Social cohesion change			1.03 (0.99, 1.07), .21	1.03 (0.99, 1.08), .17
**Neighbourhood and nativity interaction**				
Physical disorder baseline				
Native-born				Reference
Foreign-born				0.87 (0.69, 1.09), .22
Physical disorder change				
Native-born				Reference
Foreign-born				0.86 (0.68, 1.08), .19
Social cohesion baseline				
Native-born				Reference
Foreign-born				0.98 (0.87, 1.11), .75
Social cohesion change				
Native-born				Reference
Foreign-born				0.96 (0.87, 1.07), .48
Unweighted population size	5,362	5,362	5,362	5,362
Weighted population size	26,851,237	26,851,237	26,851,237	26,851,237
Model significance	*F*(1,54) = 83.92, *p* < .001	*F*(22,54) = 68.89, *p* < .001	*F*(26,54) = 58.10, *p* < .001	*F*(30,54) = 57.25, *p* < .001

Additionally, it was observed that both physical disorder at baseline (aHR = 1.15, 95% CI = 1.05–1.25, *p* < .001) and the change in physical disorder (aHR = 1.14, 95% CI = 1.06–1.22, *p* < .01) were significantly associated with an increased dementia risk. However, neither baseline neighbourhood social cohesion (aHR = 1.02, 95% CI = 0.98–1.06, *p* = .31) nor change in social cohesion (aHR = 1.03, 95% CI = 0.99–1.07, *p* = .21) was significantly associated with dementia risk.

### Moderation test

In our interaction model (Table 2, Model D), we found that neighbourhood physical disorder and social cohesion did not significantly moderate the relationship between nativity and dementia risk. In a sensitivity analysis with the listwise deletion model, physical disorder change was a significant moderator (data not shown). Compared to native-born older adults, higher physical disorder at follow-up versus baseline decreased dementia risk for foreign-born older adults (aHR = 0.78, 95% CI = 0.63–0.98, *p* = .03).

## Discussion

In our study, we observed that foreign-born older adults in the U.S. had a higher risk of developing dementia compared to their native-born counterparts. Prior research investigating the association between nativity and dementia has yielded inconsistent results. Some studies have been limited in scope, focusing on a specific race or ethnicity (Guo *et al.*, 2021; Weden *et al.*, 2017), while others have faced limitations in sample size (Kim *et al.*, 2022). However, our findings align with a recent study that analysed our same NHATS data and concluded that older immigrants had a 70% greater odds of having dementia (Franco and Choi, 2020). We expanded on their cross-sectional study using the 2011 NHATS data by using 10 years (2011–2020), and our prospective longitudinal study provides further support suggesting that foreign-born older adults may have an increased dementia risk.

One possible explanation for the heightened risk of dementia among older immigrants is their chronic exposure to stress and discrimination, which can manifest in various forms and affect multiple aspects of their lives (Higuera and Jiménez, 2023). Discrimination-induced stress inhibits their ability to establish a sense of security and stability in their host country (Higuera and Jiménez, 2023). Certain characteristics of immigrants, such as skin tone or limited language skills, which are often difficult or impossible to change, have consistently been associated with increased discrimination in social interactions (Szaflarski and Bauldry, 2019). This discriminatory treatment extends to the workplace, where immigrants, particularly Hispanic individuals, encounter limited job opportunities (Bradley-Geist and Schmidtke, 2017). They frequently find themselves in labour-intensive and hazardous positions, which not only impede upward mobility but also expose them to exploitation and the constant fear of immigration raids (Pourat *et al.*, 2021).

Discrimination has been widely linked to adverse outcomes in physical, mental and cognitive health. A recent study discovered that lifetime experiences of discrimination were associated with a 40% increased risk of developing dementia (Bancks *et al.*, 2023). Research also indicates that discrimination and dementia risk are not contingent on race or ethnicity, exposing all immigrants who face discrimination to an elevated risk (Bancks *et al.*, 2023; Dark *et al.*, 2023). Ultimately, chronic stress can contribute to the exacerbation of clinical signs commonly associated with dementia, such as high blood pressure (Dolezsar *et al.*, 2014).

Our study revealed noteworthy findings regarding foreign-born older adults and their living conditions. Firstly, respondents were significantly more likely to reside in neighbourhoods with higher levels of physical disorder at both baseline and follow-up. This trend may be attributed, in part, to a higher proportion of foreign-born individuals living in metropolitan areas, where indicators of physical disorder, such as graffiti and broken curbs, were more prevalent. Additionally, our results indicate that foreign-born older adults generally achieved lower levels of education compared to their native-born counterparts, potentially resulting in lower socio-economic status and residing in more disadvantaged neighbourhoods with higher physical disorder (Doerschuk *et al.*, 2016).

Contrary to our initial hypothesis, which proposed that foreign-born adults might be more likely to reside in ethnic enclaves that foster social connections, our findings demonstrated that the participants in our study reported lower levels of social cohesion. One plausible explanation for this discrepancy is that since foreign-born older adults were also more likely to live in neighbourhoods with higher physical disorder, the environment likewise becomes less of a conducive space for social interactions (Choi and Matz-Costa, 2017; Zahnow *et al.*, 2022). This, coupled with the challenges foreign-born older adults face in mastering second language skills, may create barriers that hinder their ability to form social connections with their neighbours (Maleku *et al.*, 2019).

These findings have significant implications for stakeholders, including healthcare professionals, who should be aware of the increased vulnerability of foreign-born older adults to dementia to ensure accurate diagnosis and appropriate care (Franco and Choi, 2020). This is especially critical given that there is currently no cure for dementia, and regular screening is essential to identify early signs of cognitive impairment for FDA-approved treatments to most effectively slow disease progression (Alzheimer’s Association, 2023). Policymakers should prioritize immigrant health by allocating resources to invest in immigrant neighbourhoods, with a focus on reducing physical disorder, which has been associated with increased dementia risk over time (Wong and Wang, 2022).

Future research is needed to explore the underlying reasons behind the observed relationship between nativity and dementia. This may include developing partnerships between patients, family members (e.g. caregiver or translator), researchers and clinicians to engage foreign-born adults in biomedical research (Wong *et al.*, 2023), which has been shown to be one of the most effective strategies to recruit and retain underrepresented populations in dementia research (Wong *et al.*, 2019). Understanding the factors that place foreign-born older adults at a higher risk of developing dementia can inform more effective prevention and intervention strategies. Additionally, studying the factors influencing the residential patterns of foreign-born older adults, including physical disorder and social cohesion in their neighbourhoods, can lead to informed policies that promote better physical, mental and cognitive health for this population.

There are several notable limitations in our study. Foreign-born is a heterogenous group, and we do not have data on the native country for respondents born outside the U.S. Similarly, we did not account for the length of residence in the U.S. since immigrating. However, we believe this may have a minimal impact given that our respondents are beneficiaries of Medicare, which requires at least 5 years of continuous residence in the U.S. before enrolment. Although all our research questions and analyses were planned before viewing the results, we did not publish a protocol. Finally, it is possible that our dementia risk estimates are biased because we censored respondents who died, instead of accounting for this competing risk. Despite these limitations, our study is an important contribution to the literature and addresses multiple knowledge gaps by investigating the relationship between nativity, neighbourhood and dementia using a nationally representative and prospective sample from a 10-year window.

## Conclusions

Our study provides valuable insights into the increased risk of dementia among older immigrants. Residing in disadvantaged neighbourhoods may contribute to increased chronic stress, highlighting the importance of public health initiatives aimed at improving living conditions. Developmental investments that enhance quality of life and promote socio-economic growth, such as the creation of parks and retail shopping centres, may be effective strategies (Zahnow *et al.*, 2022). However, further research is needed to identify the specific mechanisms underlying the increased dementia risk among older immigrants. Understanding these mechanisms will support the development of targeted interventions and action plans to reduce dementia incidence within this vulnerable population.

## Data Availability

Data sharing is not available because this study received approval to analyse sensitive data from the NHATS. Researchers interested in obtaining this data may submit a Sensitive Data Application to NHATS: https://nhats.org/researcher/data-access.
